# Editorial: Epigenetic Remodeling of Microorganisms of Pharmaceutical and Industrial Importance

**DOI:** 10.3389/fmicb.2022.887208

**Published:** 2022-04-05

**Authors:** Vijay K. Sharma, Ravindra Nath Kharwar

**Affiliations:** ^1^Agricultural Research Organization (ARO), Rishon LeZion, Israel; ^2^Mycopathology and Microbial Technology Laboratory, Centre of Advanced Study in Botany, Institute of Science, Banaras Hindu University, Varanasi, India

**Keywords:** epigenetics, epigenetic engineering, epigenetic regulators, cryptic metabolite production, secondary metabolites

Microorganisms comprise a group of highly diverse life forms on the Earth. Microbes, including bacteria and fungi, produce a vast variety of natural products that are an unparalleled resource of industrial and pharmaceutical significance. Bacterial and fungal genome sequencing disclosed the presence of numerous secondary metabolites biosynthetic gene clusters (BGC). For example, analysis of next-generation sequencing data of *Aspergillus* spp. has revealed that a single strain has far more secondary metabolites BGCs that outnumber previously isolated natural products in the laboratory (Khaldi et al., 2010; Sanchez et al., 2012). These silent (cryptic) gene clusters harbor a treasure of novel bioactive metabolites for drug discovery. However, under standard laboratory conditions, only a portion of these compounds are accessible. Under normal conditions, some BGCs remain silent or expressed at low levels (Skellam, 2019; Wu et al., 2020). Gene expression can be triggered by changing cultivation parameters or applying physical, biological or chemical stresses (Scherlach and Hertweck, 2009). These silent genes could be activated by employing epigenetic manipulation approaches. Epigenetics describes the heritable changes in gene expression that occur without a change in the genome sequence. These epigenetic changes of the DNA strands are reversible, enabling adaptation and modulation of gene(s) expression in fluctuating environments.

In recent years, such epigenetic modulation has led to the discovery of several newly identified natural biomolecules. Epigenetic manipulations include gene knockouts that alter the expression and functionality of specific enzymes involved in creating and modulating DNA methylation patterns. Common molecular modifications that form the basis of epigenetic gene regulation include DNA methylation, chromatin remodeling, covalent histone modification, the localization of histone variants and feedback loops (Akone et al., 2019; Poças-Fonseca et al., 2020) (Figure 1). Consequently, DNA methyltransferase inhibitors and/or histone deacetylase inhibitors frequently target the genes knockout. Treatments of bacteria and fungi with DNA methyltransferase (DNMT) inhibitors like 5-azacytidine, procaine, procainamide, and hydralazine, and histone deacetylase (HDAC) inhibitors like sodium butyrate, suberoylanilide hydroxamic acid (SAHA) and valproic acid have been reported to induce the cryptic genes or activation of biosynthetic pathways of secondary metabolites. In addition to genetic modulation, a number of recent studies have suggested that various active food components present in dietary ingredients like turmeric, grapes, or green tea may also stimulate epigenetic changes (Sharma et al., 2017; Evans et al., 2020; Mishra et al., 2020). Pathogen-triggered epigenetic changes may also alter host cell functions, either to promote host defense or to favor pathogen persistence. Epigenetic mechanisms are known to repress and control expression of genes involved in pathogenicity (Tannous et al., 2020). Thus, epigenetic regulations have the potential application in controlling pathogenesis and post-harvest plant pathogens. Epigenetic perturbation of gene clusters leverages microorganisms for biosynthesizing new chemical entities of pharmaceutical and industrial importance. Further, epigenetic manipulations are also known to affect the metabolite pathways such as polyketide biosynthesis, induced production of cryptic compounds, restoration of attenuated compounds, and the production of host origin bioactive compounds.

**Figure 1:**
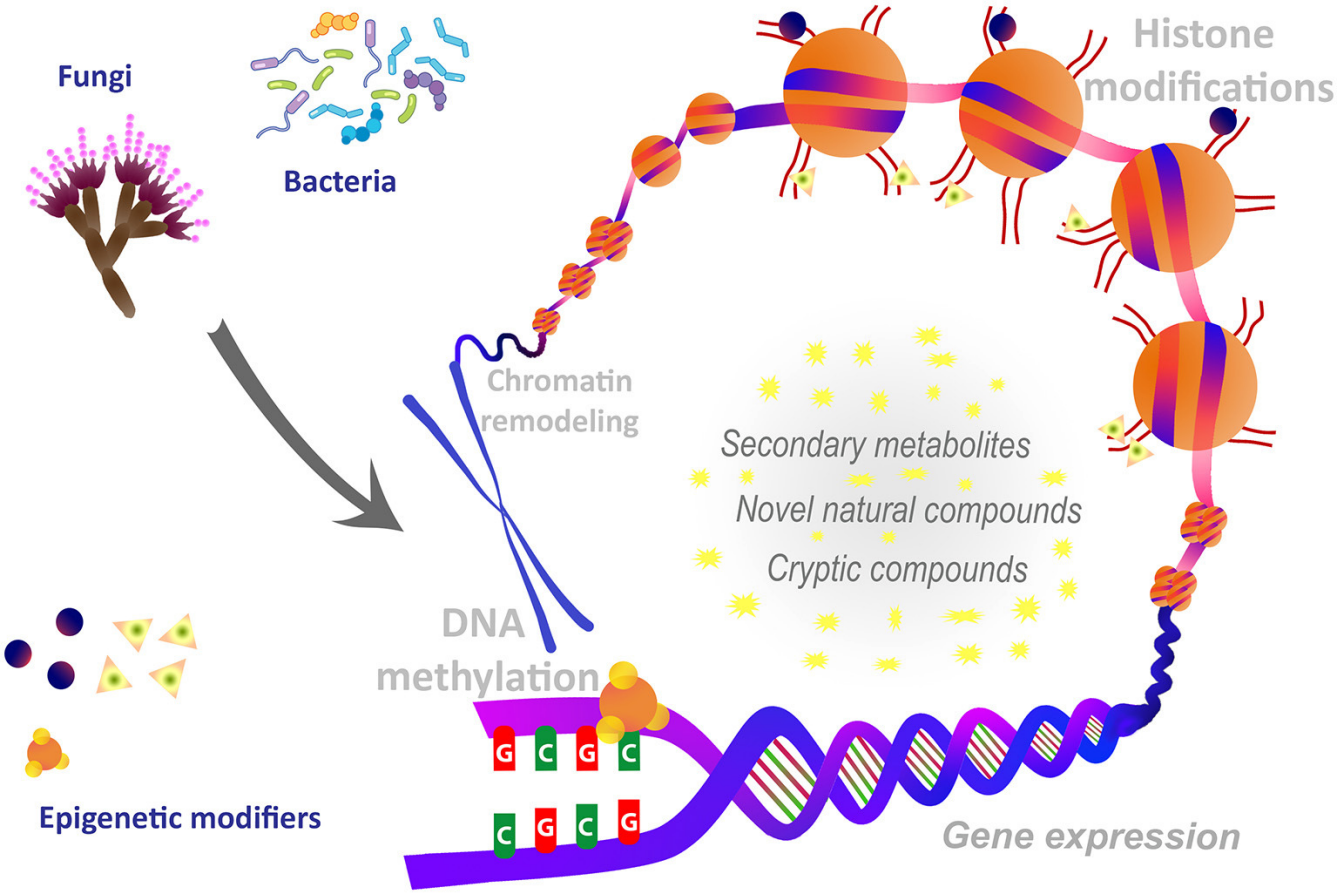
Epigenetic tailoring in microorganisms.

In a review, Pillay et al. draw attention to the regulatory circuits in fungi governing the expression of secondary metabolites. They discussed chromatin, and its role in controlling gene expression, as well as the response to epigenetic treatment at the metabolome level. A brief account of small molecular modifiers, their action mechanisms, target sites, advantages, and disadvantages are discussed. Furthermore, they illustrated that genetic deletion or epigenetic inhibition of histone deacetylases does not necessarily lead to the overexpression or induction of silent BGCs. Rather, the response is more complex and often leads to differential expression of secondary metabolites. In a mini-review, Chaturvedi et al. outlined key epigenetic modifications such as DNA methylation, chromatin remodeling, RNA interference, and their impact on gene expression in enhancing the production of secondary metabolites (SMs) in microorganisms. Fungi, especially those that are associated with endosymbiotic relationships with plants, are now acknowledged as future sources of novel natural compounds. Nishad et al. investigated the efficacy of BRD4770, a novel histone methyltransferase inhibitor, in the epigenetic manipulation of silent BGC in fungal endophytes. *Diaporthe longicolla*, an endophyte isolated from the stem of *Saraca asoca*, was treated with different concentrations of BRD4770, which induced the bioactive compounds with enhanced antibacterial and antioxidant activities. With the help of GC-MS and LC-ESI-MS/MS, the induced compounds were identified as berberine (antibacterial), caffeine, and theobromine (antioxidant). Ramesha et al. evaluated six known modifiers, *viz*., two DNMT inhibitors (5-azacytidine and hydralazine hydrochloride), a sirtuin activator (quercetin) and three HDAC inhibitors (SAHA, sodium butyrate, and valproic acid) for epigenetic modulation in the endophytic fungus *Nigrospora sphaerica*. He concluded that compared to DNMT treatments, HDAC treatments induced more cryptic metabolites. Vieira et al. evaluated several cultivation methods using different culture media and chemical elicitors to increase the production of epoxyketone peptide by an actinobacteria, *Streptomyces* sp. BRA-346, isolated from a Brazilian endemic tunicate, *Euherdmania* sp. Genome sequencing and mining of BRA-346 showed that it has a wide range of biosynthetic capacity. Under laboratory conditions, ampicillin increased epoxyketone and downregulated macrolides, terpenes, and diketopiperazines/alkaloids, while procaine had the opposite effect, downregulating epoxyketone peptides and upregulating the three other major groups (macrolides, terpenes, and diketopiperazines/alkaloids). A single BGC was related to the production of the target epoxyketone peptides by BRA-346. By cloning the BRA-346 epn/tmc BGC into *S. coelicolor* M1146, Vieira et al. succeeded in getting epoxyketone peptides and several congeners, including eponemycin. This work is a comprehensive model of exploring natural products in a newly isolated *Streptomyces* strain from a unique niche. It combines ultra-modern biotechnological approaches to reveal the chemistry and identify BGCs associated with epoxyketone peptide production, furthering possible strategies to improve its production.

Thus, this Research Topic brings new insights into the epigenetic modulation of bacteria and fungi using epigenetic modifiers to improve the production of secondary metabolites and/or cryptic metabolites that have potential applications in pharmaceutics and industries. As of now, limited studies are available on the epigenetic modulation of microorganisms. We hope this small collection of articles will encourage more researchers to work on this subject of increasing importance and interest. Future studies and discussions are required not only in respect of applications but also related to the mechanisms of epigenetic modulation.

## Author Contributions

Both authors drafted the content of this document, revised, and approved the final version of the editorial text for submission.

## Conflict of Interest

The authors declare that the research was conducted in the absence of any commercial or financial relationships that could be construed as a potential conflict of interest.

## Publisher's Note

All claims expressed in this article are solely those of the authors and do not necessarily represent those of their affiliated organizations, or those of the publisher, the editors and the reviewers. Any product that may be evaluated in this article, or claim that may be made by its manufacturer, is not guaranteed or endorsed by the publisher.
